# Effect of Osmotic Dehydration on Physico-Chemical Characteristics, Bioactive Compounds and Volatiles Profile of *Diospyros kaki* Subjected to Different Drying Methods

**DOI:** 10.3390/foods14101727

**Published:** 2025-05-13

**Authors:** Cecília Morais Santana Matos, Mônica Silva de Jesus, Augusto de Souza da Silva, Nayjara Carvalho Gualberto, Hannah Caroline Santos Araujo, Rafael Donizete Dutra Sandes, Raquel Anne Ribeiro dos Santos, Maria Terezinha Santos Leite Neta, Narendra Narain

**Affiliations:** 1 Laboratory of Flavor and Chromatographic Analysis, Federal University of Sergipe, Av. Marcelo Deda Chagas, s/n, Jardim Rosa Elze, São Cristóvão 49100-000, SE, Brazil; c.moraismatos@gmail.com (C.M.S.M.); monicasj.sst@gmail.com (M.S.d.J.); augustoss@hotmail.com.br (A.d.S.d.S.); nayjaracarvalho@gmail.com (N.C.G.); hcarol197@gmail.com (H.C.S.A.); rafael.donizete.dutra@gmail.com (R.D.D.S.); terezinhaleite@gmail.com (M.T.S.L.N.); 2 Federal Institute of Education, Science and Technology of Sergipe, Rod. BR 101, Km 96, s/n, Quissamã, São Cristóvão 49100-000, SE, Brazil; eng.raquelanne@gmail.com; 3 Department of Food Technology, Federal University of Sergipe, Av. Marcelo Déda Chagas, s/n, Jardim Rosa Elze, São Cristóvão 49100-000, SE, Brazil

**Keywords:** persimmon, osmotic dehydration, bioactive compounds, volatile compounds

## Abstract

The seasonality of *Diospyros kaki* limits its consumption, making it increasingly necessary to develop products that allow it to be available all year round. Therefore, the aim of this study was to optimize the osmotic dehydration process and to evaluate the changes in the physico-chemical, bioactive and volatile composition of persimmon subjected to drying. A central composite rotatable design was used with the independent variables as sucrose concentration (%) and immersion time (min). The desirability function for sucrose solution concentration and immersion time was 29.5% for 60 min for hot air oven drying and 38% for 29 min for freeze drying. The dehydrated fruit treated with osmotic pretreatment showed better responses in the quantification of bioactive compounds and retention of volatile compounds. Compounds such as nonanal and 6-methyl-5-hepten-2-one were identified in all the dehydrated products and the OD had a positive influence on their retention, especially for the lyophilized samples. Thus, it is clear that osmotic pretreatment is an ally in protecting the physico-chemical and chemical characteristics of the fruit when it undergoes a drying process, especially with regard to bioactive and volatile compounds.

## 1. Introduction

Persimmon (*Diospyros kaki*) is a fruit originating from China that initially spread to Japan and South Korea and is currently cultivated in numerous other countries in the world, including Brazil [1,2]. Persimmon is a tasty, attractive fruit with high nutritional value, proteins, fiber, minerals, carbohydrates, in addition to being rich in biologically active substances, including phenolic compounds, various vitamins, carotenoids and tannins, which have numerous physiological qualities, including protection against oxidative stress and carcinogenic processes [3,4].

Due to its high perishability and rich composition, there is a need for technological alternatives to make better use of it [1]. In order to increase the longevity of persimmons, it is necessary to eliminate or reduce their water content, and drying is the most effective technique for this purpose and to extend the shelf life of foods [5,6]. This can be achieved by using heat under controlled conditions to evaporate most of the water present in the fruit or by employing the freeze-drying process in which the frozen water is sublimated to approximately −50 °C under vacuum [5,7]. The hot air-drying process is still the most widely used technique for dehydrating food, although it still results in numerous undesirable changes to the food [5,8]. Freeze-drying, on the other hand, is widely recognized as the most effective drying method for maintaining the inherent qualities of the raw material and retaining sensitive substances such as bioactive and volatile compounds [9,10].

Osmotic dehydration (OD) is a low-cost and complex non-thermal dehydration process that has been used as a pretreatment for other drying methods as an alternative to maintain the properties and characteristics of the products and reduce the operating cost of conventional drying [11,12]. This process consists of immersing the food in a hypertonic solution, so the free water present in the food is transferred to the solution due to the semi-permeability of the cell membrane, and in the opposite direction, the solute used in the hypertonic solution flows into the food itself in small proportions [13,14].

OD maintains the physicochemical and functional properties of the fruit, preserves the integral and organoleptic qualities of the products, prevents oxidative browning and the loss of volatiles, and promotes the extension of the shelf life of dehydrated products [14,15]. OD is commonly combined with other treatments, such as ultrasound, whose cavitation force principle contributes to increased mass transfer during the OD process, thus enhancing the impacts of the treatment on the food [12,16]. Several OD studies have been conducted on fruits such as mango [16,17,18], kiwi [19,20], plum [21], strawberry [22], apple [23], peach [24], among others.

Despite being an attractive alternative for fruit processing, few studies involving the application of OD in persimmons are available in the literature, and these are mainly limited to the use of OD as a pretreatment which can also be used in conjunction with ultrasound before hot air-drying at different temperatures and aimed at evaluating the drying characteristics and/or quality of the persimmon [25,26,27]. Thus, the objective of this study was to use OD as a pretreatment for both oven air-drying and freeze-drying of persimmons, and to evaluate the influence of these processes on the physico-chemical characteristics and bioactive and volatile compounds in dehydrated products.

## 2. Materials and Methods

### 2.1. Materials

The persimmons fruits (*Diospyros kaki* Thunb.) with uniform color, shape, size, firmness, absence of injuries or diseases and in the mature stage of ripeness were obtained from the Aracaju Supply Center (CEASA). The purchased persimmons were washed and sanitized with 200 ppm of chlorine for 15 min, manually peeled and sliced into uniform pieces of about 3 cm^2^. The samples were divided into a control group, that is, samples that were not subjected to osmotic dehydration pretreatment; and samples subjected to osmotic dehydration before drying in an oven with air circulation or by freeze-drying.

### 2.2. Chemicals, Reagents and Encapsulating Agent

Ferric chloride was obtained from Vetec Química Fina Ltd. (Duque de Caxias, Rio de Janeiro, Brazil). Sodium acetate and monobasic sodium phosphate were obtained from Dinâmica Química Contemporânea Ltd. (Indaiatuba, Brazil). Ethanol (HPLC grade) was obtained from Tedia High Purity Solvents (Porto Alegre, Brazil). Acetone (HPLC grade) was obtained from J.T. Baker (Aparecida de Goiânia, Goiás, Brazil). Ascorbic acid, 2,2′-azino-bis (3-ethylbenzothiazoline-6-sulfonic acid) (ABTS), Folin–Ciocalteu phenol reagent, gallic acid, quercetin, 2,4,6-tripyridyl-s-triazine (TPTZ) 6-hydroxy-2,5,7,8-tetramethylchroman-2-carboxylic acid (Trolox), analytical standards of the organic acids (acetic acid, oxalic acid, quinic acid, L-ascorbic acid, fumaric acid, citric acid, and succinic acid), 2-decanone and C8–C40 n-alkanes standard were obtained from Sigma-Aldrich (St. Louis, MO, USA).

### 2.3. Optimization of Osmotic Dehydration (OD) Process

The experimental design was developed using a central rotational composite design (CCRD) type 2^2^, with 4 axial points and 3 repetitions at the central point, totaling 11 assays. The 2 independent variables in the experimental design were chosen as sucrose concentration (X_1_, 10–40%) and immersion time of persimmons in the sucrose solution (X_2_, 20–60 min) (Table 1). The intervals of the variables were defined according to Demiray and Tulek [26] and Bozkir et al. [27] with adaptations. The ultrasound-assisted OD process was performed in an ultrasonic bath (Unique, USC-1400A, Campinas, Brazil) with a fixed sonication power of 135 W, frequency of 40 kHz and temperature of 25 °C. Later, the samples were subjected to hot air-drying or freeze-drying processes.

The influences of the independent variables of the osmotic dehydration experimental design were evaluated based on important characteristics for dehydrated fruits such as physico-chemical characteristics (aw, moisture, pH, acidity and color), content of bioactive compounds (carotenoids, phenolics and flavonoids) and antioxidant activity (ABTS and FRAP).

### 2.4. Drying Experiments

#### 2.4.1. Hot Air-Drying (HAD)

The samples (control and osmotic dehydration assays) were placed in trays and subjected to a drying process in a forced air circulation oven (Marconi, MA 035, São Paulo, Brazil) at a fixed temperature of 35 °C until constant moisture content was obtained.

#### 2.4.2. Freeze Drying (FD)

The samples (control and osmotic dehydration assays) were placed in acrylic trays with 12.5 cm in diameter and 1.0 cm in height, previously frozen in a vertical freezer (−18 °C) for 24 h and subjected to the drying process in a bench freeze dryer (Christ, Alpha 1–2 LD plus, Osterode am Harz, Germany) at a temperature of −50 °C, pressure of 6.11 mbar and vacuum of 0.42 mbar for 48 h.

### 2.5. Physico-Chemical Analysis

The moisture, water activity and pH were determined using an infrared moisture analyzer (Gehaka, IV2500, São Paulo, Brazil), water activity meter (Aqualab, 4TEV, Aparecida, Brazil) and digital potentiometer (Hanna, HI 2221, Amorim, Portugal), respectively. Colorimetric analysis was performed using a portable spectrophotometer (Konica Minolta, CM-700d, Tokyo, Japan) and expressed in the CIELab color space (parameters L*, a*, b*). The titratable acidity was determined by titration with 0.1 N sodium hydroxide (NaOH) solution. All methodologies presented in this topic followed the protocols proposed by the Instituto Adolfo Lutz [28] and the Association of Official Analytical Chemists [29]. Analysis was performed in triplicate and results were expressed as mean ± standard deviation values.

### 2.6. Determination of Bioactive Compounds and Antioxidant Capacity

#### 2.6.1. Total Carotenoids (TCs)

The TC content was determined according to the methodology of Lichtenthaler [30]. The absorbances were measured in a spectrophotometer (Molecular Devices, CA, USA; Spectra Max M2) at 646.8, 663.2 and 470 nm, and the results expressed in µg/g. The concentration was estimated according to Equations (1)–(3):

Ca (chlorophyll a) = 12.25 × A663.2 − 2.79 × A646.8(1)

Cb (chlorophyll b) = 21.50 × A646.8 − 5.10 × A663.2(2)

Carotenoids = {100 × A470 − [(1.82 × Ca) − (104.96 × Cb)]}/198(3)

#### 2.6.2. Preparation of the Extracts

To obtain the extracts, 5 g of sample was mixed with 50 mL of 70% (*v*/*v*) ethanol. The samples were then sonicated in an ultrasound bath (Unique, USC-1400A, 40 kHz) at 30 °C/60 min. The extracts were vacuum-filtered and concentrated in a rotary evaporator (Buchi model R-3, São Paulo, Brazil) at 40 °C and evaluated for the contents of phenolic compounds and flavonoids.

#### 2.6.3. Determination of Total Phenolic Compounds (TPCs)

The TPCs were determined using the conventional Folin–Ciocalteu spectrophotometric procedure [31]. The absorbance was measured using a spectrophotometer at 725 nm, and the results were expressed in mg of gallic acid equivalent (GAE)/100 g of sample calculated using a gallic acid standard curve, constructed with concentrations varying from 20 to 160 μg/L.

#### 2.6.4. Determination of Total Flavonoids (TFs)

The TFs were determined according to the method of Karaman et al. [10]. The absorbance of the solutions was recorded in a spectrophotometer at a wavelength of 415 nm. A standard curve of quercetin (0.0–100 mg/L) was constructed for quantitative determinations, and the results were expressed as mg quercetin equivalents (QE)/100 g.

#### 2.6.5. Antioxidant Capacity Assays

The extracts produced from the dehydrated persimmons were evaluated for antioxidant activity using the ABTS^•+^ radical capture assay and also for ferric reducing antioxidant power (FRAP).

The ABTS analysis was carried out adopting the methodology proposed by Rufino et al. [32]. In an assay tube containing 3 mL of activated ABTS^•±^ radical, 30 µL of the extract was added and vortexed. After 6 min in a dark environment, the absorbance was read at 734 nm using a spectrophotometer. A standard curve of trolox was used at concentrations of 100–2000 μmol/L and the results were expressed as µmol of Trolox equivalent /100 g of sample.

The FRAP assay was performed according to the methodology of Rufino et al. [33]. In an assay tube, 90 μL extract, 270 μL distilled water and 2.7 mL FRAP reagent at 37 °C were homogenized. After 30 min in the dark, these were kept at 37 °C and later the absorbance was read at 595 nm in a spectrophotometer. The antioxidant activity was calculated from a standard curve constructed of ferrous sulphate (500–2000 μmol/L) and expressed in μmol ferrous sulphate/100 g of sample.

### 2.7. Identification and Quantification of Organic Acids by HPLC-DAD

The extraction of organic acids followed the methodology proposed by Lee [34] with modifications. A total of 1 g of sample and 9 mL of mobile phase (monobasic sodium phosphate (0.01 M)), at pH 2.5, were agitated in a shaker (SPLABOR, SP-222, São Paulo, Brazil) at 120 rpm for 2 h. Later, the mixture was centrifuged (Eppendorf, model 5810 R, São Paulo, Brazil) at 12,000 rpm for 15 min and the supernatant filtered on PVDF membrane (0.45 μm).

The extracts obtained were analyzed using High-Performance Liquid Chromatography (HPLC) with UV-DAD (Diode Array Detection system; Shimadzu Corporation, Kyoto, Japan). The organic acids were separated on a column (Phenomenex C18; 250 × 4.6 mm; 5 μm) maintained at 40 °C. The injection volume was 5 µL, and an isocratic elution was performed with a mobile phase at a flow rate of 1 mL/min. For quantification, external calibration curves were constructed for each analytical standard. The results were expressed in mg/100 g of the sample.

### 2.8. Determination of Volatile Compounds by Solid Phase Micro Extraction (SPME) and GC-MS

The volatile compounds from persimmon pulp and dehydrated persimmon were extracted following the methodology of Hung et al. [35], with adaptations based on the methodology of Cheong et al. [36]. The sample (2 g) was transferred to a 20 mL vial containing 0.2 g of sodium chloride (NaCl), 10 mL of deionized water and a magnetic stir bar. The vial was sealed with a silicone septum and immersed in a water bath at a constant temperature of 25 °C. After 15 min of agitation (to reach equilibrium in the system), a polydimethylsiloxane/divinylbenzene fiber (65 μm, PDMS/DVB; Supelco, Bellefonte, PA, USA) was exposed to the vial headspace for 30 min. During the extraction process, the sample remained stirred at a temperature of 25 °C. The extraction was carried out in triplicate.

Analysis of the compounds was performed on a gas chromatograph (Agilent Model 7890A, Santa Clara, CA, USA) coupled to a mass spectrometer (Agilent Model 7000, Santa Clara, CA, USA). The GC was equipped with a HP-5MS capillary column (30 m length × 0.25 mm i.d. × 0.25 μm film thickness, Agilent). Helium was used as carrier gas at a flow rate of 1.0 mL/min. with injection in splitless mode. The injector temperature was maintained at 250 °C, and the oven operating temperature range was from 40 to 250 °C: initial temperature of 40 °C (maintained for 3 min) raised at a rate of 3 °C/min until 130 °C (maintained for 10 min), and then raised to 250 °C at 10 °C/min, remaining at this temperature for 3 min.

The electron impact ionization energy (EI) was 70 eV and the scanning range was between 40 *m*/*z* and 400 *m*/*z*. The mass spectra obtained were compared with the mass spectra in the mass spectral database version NIST 13 (National Institute of Standards and Technology, Washington, DC, USA).

### 2.9. Quantification of Volatile Compounds

The quantification of the identified compounds was performed based on the analysis of the 2-decanone standard (final concentration of 500 ppb; Sigma-Aldrich) performed under analytical conditions identical to the samples. A response factor equal to 1.0 was considered between the relative peak areas of each compound and the peak area of the standard. The concentrations of the compounds were expressed in μg/kg.

### 2.10. Statistical Analysis

The data obtained were subjected to analysis of variance (ANOVA), Pareto Diagrams, Response Surface Methodology and Desirability function using the Statistica version 12.5 software (StatSoft Inc., Tulsa, OK, USA).

## 3. Results and Discussion

### 3.1. Optimization of Persimmon Osmotic Dehydration Process with Hot Air-Drying

**Figure 1:**
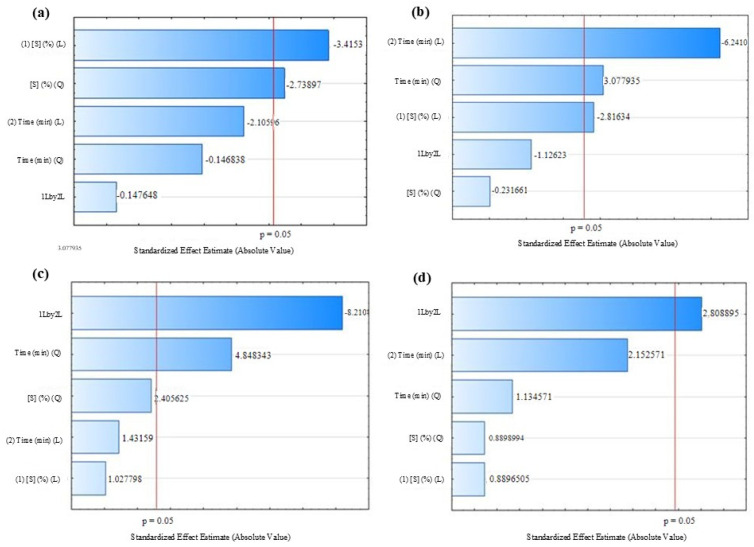
Effect of the independent variables in OD-HAD process on the response variables: (**a**) aw; (**b**) moisture content; (**c**) concentration of total flavonoids; (**d**) antioxidant activity by ABTS.

Table 2 presents the results obtained for the response variables that suffered significant influence (*p* ≤ 0.05), according to the Pareto diagrams (Figure 1), based on the combinations between the levels of the independent variables indicated in the experimental design.

The lowest aw (0.5302) and moisture (9.91%) contents were observed in the sample of assay 6; that is, when the highest sucrose concentration (40%) and the central point of the immersion time were used. Generally, the sucrose concentration used for osmotic dehydration of fruits is between 40 and 55% [37]. Very low concentrations of the osmotic solution can result in low osmotic pressure and insufficient driving force to remove water from the materials into the solution, whereas increasing the sucrose concentration results not only in a faster dehydration rate but also in a lower equilibrium water content or greater water loss [38,39].

The highest TF concentration and antioxidant activity by the ABTS method were observed in assays 2 and 4, respectively. Pareto diagrams showed that the linear term (L) of sucrose concentration had a significant negative effect (*p* ≤ 0.05) on aw and moisture content (Figure 1a,b). The interaction between the linear terms of sucrose concentration and immersion time had a significant negative effect (*p* ≤ 0.05) on total flavonoids concentration and a positive effect (*p* ≤ 0.05) on antioxidant activity by ABTS (Figure 1c,d). The quadratic term of immersion time showed a significant positive effect on both moisture content and flavonoids concentration (Figure 1b,c).

The fitted models for aw (Y_1_), moisture (Y_2_), flavonoid concentration (Y_3_) and antioxidant activity by ABTS (Y_4_) are described by Equations (4)–(7), respectively, while X_1_ and X_2_ refer to sucrose concentration (%) and immersion time (min), respectively. The accuracy of the models can be inferred from the coefficient of determination (R^2^) whose values were 0.8282, 0.9212, 0.9497 and 0.7493 for Y_1_, Y_2_, Y_3_ and Y_4_, respectively.

Y_1_ = 0.55 − 0.0048X_1_ − 0.0039X_1_^2^ (4)

Y_2_ = 10.90 − 0.40X_1_ − 0.89X2 + 0.52X_2_^2^ (5)

Y_3_ = 255.95 + 0.024X_2_^2^ − 0.013X_1_X_2_ (6)

Y_4_ = 76247.78 + 13619.23X_1_X_2_ (7)

**Figure 2:**
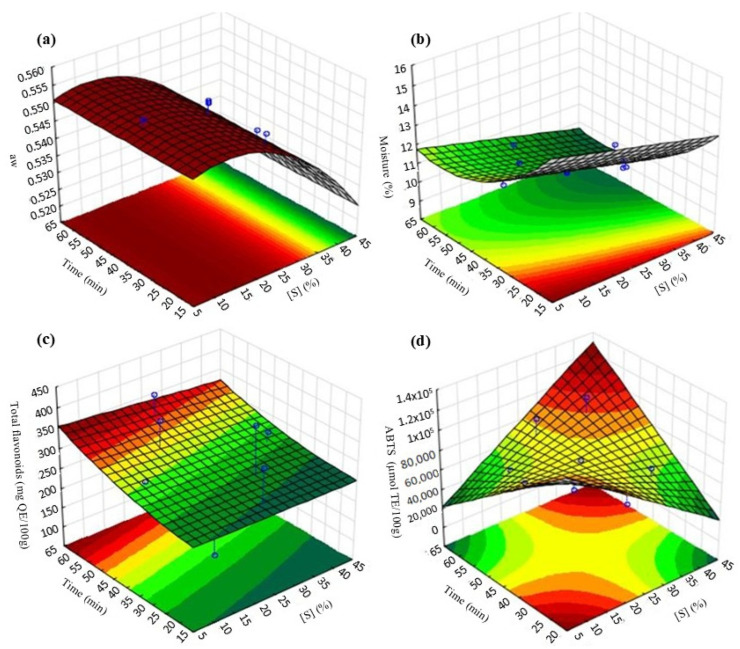
Response surfaces for the effects of the independent variables of OD-HAD process: (**a**) aw; (**b**) moisture content; (**c**) concentration of total flavonoids; (**d**) antioxidant activity by ABTS.

Response surface plots were generated to visualize the combined effect of two variables on a specific response (Figure 2), and these demonstrated that the optimum region for obtaining lower water activity values was around 10–30% sucrose (Figure 2a). Lower moisture contents were observed in the range of 15–30 min of immersion (Figure 2b); at longer immersion times, the TF content increases (Figure 2c), and higher antioxidant activity by ABTS was obtained at higher immersion times and sucrose concentrations (Figure 2d).

### 3.2. Optimization of Persimmon Osmotic Dehydration Process with Freeze-Drying

**Figure 3:**
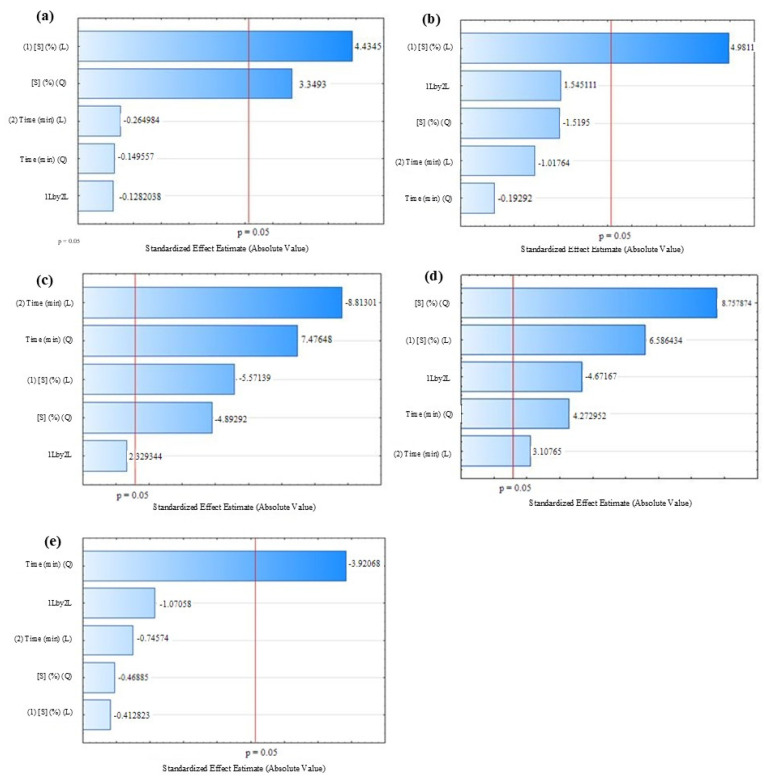
Effect of the independent variables in OD-FD process on the response variables: (**a**) concentration of total phenolics; (**b**) total flavonoids; (**c**) total carotenoids; (**d**) antioxidant activity by FRAP; and (**e**) ABTS.

Table 3 presents the results obtained for the response variables that were significantly influenced (*p* ≤ 0.05), according to the Pareto diagrams (Figure 3), based on the combinations indicated in the experimental design. The conditions of the OD-FD process significantly influenced the concentrations of the bioactive compounds and both antioxidant activities.

As can be observed (Table 3), assay 6 (highest sucrose concentration and central time point) resulted in the highest TPC and TF concentrations, and highest antioxidant activity by FRAP. Regarding the TC content and antioxidant activity by ABTS, these were higher in trials 7 and 10, respectively. Pareto diagrams showed that the linear term (L) of sucrose concentration had a significant positive effect (*p* ≤ 0.05) on TPC and TF concentration and on antioxidant activity by FRAP (Figure 3a,b, and d, respectively). The linear (L) and quadratic (Q) terms of sucrose concentration and linear (L) of immersion time had a significant negative effect (*p* ≤ 0.05) for TC (Figure 3c). The interaction between the linear terms of sucrose concentration and immersion time had a significant negative effect (*p* ≤ 0.05) for antioxidant activity by FRAP (Figure 3d).

The regression models for the response variables generated by the values of the independent variables are expressed in Equations (8)–(12), where Y_1_, Y_2_, Y_3_, Y_4_ and Y_5_ correspond, respectively, to TPC, TF, TC and antioxidant activity by FRAP and ABTS.

Y_1_ = 1041.36 − 87.95X_1_ + 2.46X_1_^2^ (8)

Y_2_ = 428.12 + 24.08X_1_ (9)

Y_3_ = 55.07 + 0.87X_1_ − 0.02X_1_^2^ − 1.95X2 + 0.021X_2_^2^ (10)

Y_4_ = −3464.28 + 108.25X_1_ + 8.50X_1_^2^ + 120.58X_2_ + 2.39X_2_^2^-11.59X_1_X_2_ (11)

Y_5_ = 148935.03 − 5.77X_2_^2^ (12)

**Figure 4:**
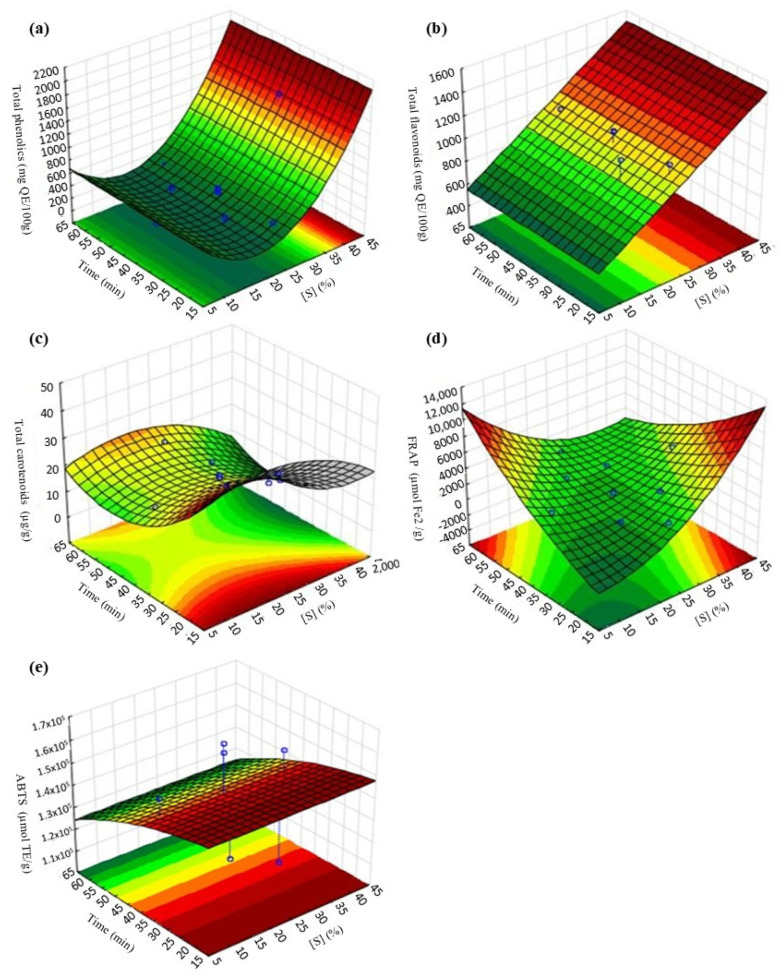
Response surfaces for the effects of the independent variables of the OD-FD process on: (**a**) concentration of total phenolics; (**b**) total flavonoids; (**c**) total carotenoids; (**d**) antioxidant activity by FRAP; and (**e**) ABTS.

The coefficient of determination (R_2_) of the models generated for Y_1_ (0.8694), Y_2_ (0.8600), Y_3_ (0.9795), Y_4_ (0.9682) and Y_5_ (0.7867) indicated good predictive capacity, mainly for the response variables Y_3_ and Y_5_. The response surfaces generated from the models are presented in Figure 4. Regarding phenolic compounds and flavonoids, the concentrations were higher when higher sucrose concentrations were used (Figure 4a,b); lower carotenoids content was obtained at higher immersion times (Figure 4c); higher antioxidant activity by FRAP was obtained at higher time and sucrose concentration (Figure 4d), and the longer the immersion time, the lower the antioxidant activity by ABTS (Figure 4e).

### 3.3. Desirability Function

**Figure 5:**
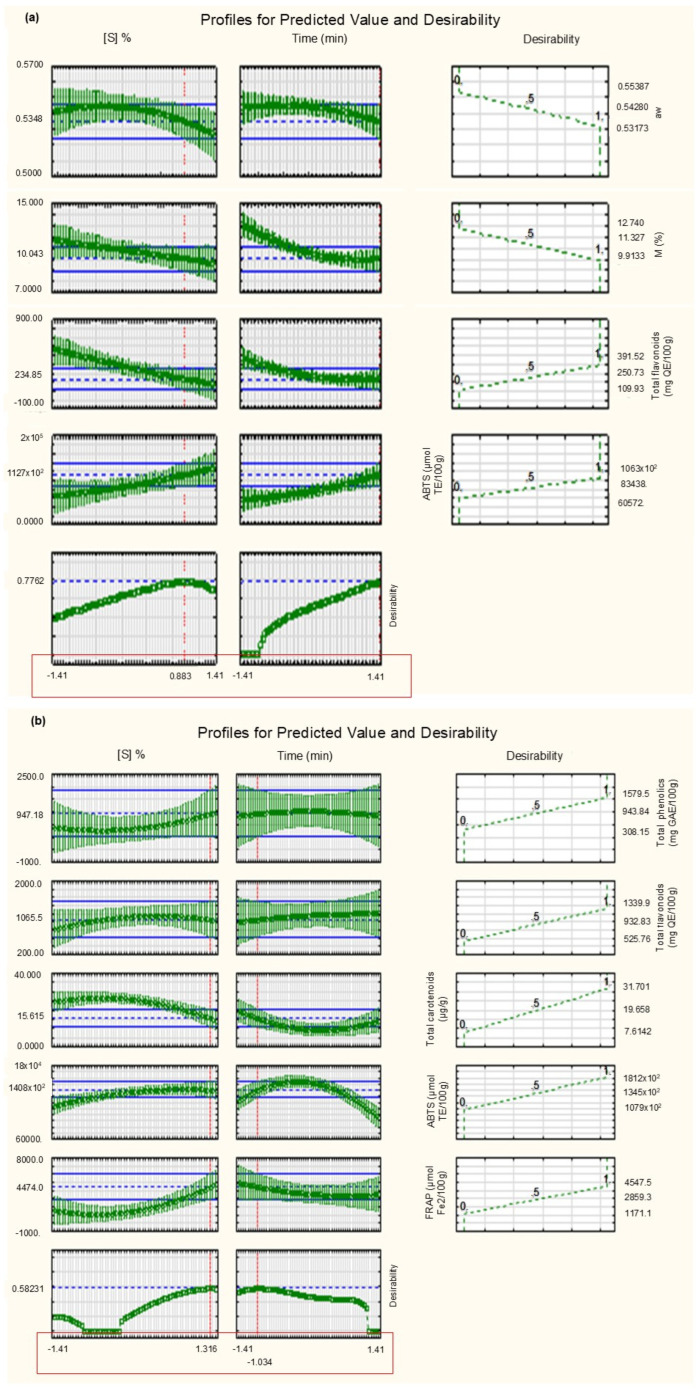
Desirability function for response variations as a function of sucrose concentration and immersion time of the osmotic dehydration process followed by hot air drying (**a**) and freeze-drying (**b**).

The Desirability function proposed by Derringer and Suich [40] is a statistical tool commonly used to optimize processes with multiple responses, transforming individual factors into their associated desirability. Figure 5 presents the global Desirability function of the data, showing the optimization conditions of the osmotically dehydrated persimmon production process and subsequent HAD or FD, respectively.

The Desirability function demonstrated that the concentration of total flavonoids and antioxidant activity by the ABTS method are maximized and the aw and moisture are minimized when adopting the condition of 29.5% concentration of the sucrose solution and 60 min with subsequent oven drying of the persimmons. As for persimmons subjected to the FD process, the variable responses (TPC, TF, TC and antioxidant activity by the ABTS and FRAP methods) are maximized when using the condition of 38% concentration of the sucrose solution and 29 min for the immersion time. A new assay was carried out to validate the conditions predicted by the Desirability function and the results are presented in Table 4.

The coefficients of variation ranged from 0.84% to 13.62%. These results demonstrate the validity of using the experimental design and the desirability function in the osmotic dehydration process and that the regression models were satisfactory for predicting the response variables.

### 3.4. Organic Acids in Dehydrated Persimmons

Organic acids were determined in: control persimmon (without osmotic dehydration) subjected to hot air-drying (C-HAD), persimmon subjected to osmotic dehydration (best conditions of the experimental design) followed by hot air-drying (OD-HAD), control persimmon (without osmotic dehydration) subjected to freeze-drying (C-FD) and persimmon subjected to osmotic dehydration (best conditions of the experimental design) followed by freeze-drying (OD-FD). A total of seven organic acids (Table 5) were identified and quantified among the tested samples.

Among the acids identified in the present study, citric, fumaric and succinic acids are some of the main organic acids identified in persimmon and reported in earlier publications [2,41].

The presence of fumaric acid has already been detected in some studies and its concentration quantified in persimmons [2,42]. However, in the present study, its presence was detected only in samples subjected to the HAD process. Gökmen and Acar [43], when evaluating the concentration of fumaric acid in apple juices, observed that small concentrations of this acid can be formed during the application of a heat treatment, such as evaporation.

The presence of oxalic acid was observed in all samples but in significantly different concentrations (*p* ≤ 0.05). Oxalic acid is one of several organic acids with potential health benefits, present in fruits and vegetables. Nguyễn and Savage [44] found a concentration of 0.074 g/kg of oxalic acid in ripe persimmon. Oxalic, acetic, ascorbic and citric acids were identified in persimmon samples grown in different regions of Korea, and their concentrations varied between 58.37 and 356.60 mg/kg [45].

Ascorbic acid was identified only in FD samples. According to Yin et al. [46], this acid is easily degraded in processes involving temperature, which may justify the fact that it was not quantified in in samples subjected to HAD but rather in FD samples, as this process occurs at low temperatures.

Citric, quinic and succinic acids were identified in persimmon samples at different stages of ripeness [47]. The concentration of citric acid in different persimmon cultivars at mature stage varied between 196 and 701 mg/kg [2]. Bian et al. [48] observed concentrations varying between 67.3 and 130.3 mg/100 g for different cultivars of astringent persimmons grown in different regions, and these values being higher than those observed in the present study.

Regarding succinic acid, it was observed that samples submitted to the OD process presented higher concentrations of this acid compared to their respective controls. It was also possible to observe that the FD samples presented lower concentrations compared to the samples submitted to HAD. Ardestani et al. [49] studied the volatiles profile of barberries submitted to various processes and observed that exposure to freezing temperatures can induce a decrease in the content of organic acids, such as succinic acid.

In general, organic acids play an important role as an indicator of fruit quality; for example, it is estimated that a higher proportion between sugars and organic acids leads to a better perception of sweet flavor [1,4].

Zhao et al. [18] evaluated the influence of different osmotic solution pretreatments on the phenolic compounds of frozen mangoes and observed that the osmotic treatment had a different influence on the concentration of some compounds present in the samples. The authors indicated that the reduction in gallic acid concentration may be related to leaching into the hypertonic solution during osmotic pretreatment or the occurrence of other chemical and biochemical reactions, such as chemical degradation and biosynthesis. As for the higher values of p-hydroxybenzoic acid observed, the authors explained that these may be related to the induction of metabolic synthesis due to osmotic stress.

### 3.5. Volatile Compounds in Dehydrated Persimmons

The volatile compounds identified in C-HAD and OD-HAD, C-FD and OD-FD are presented in Table 6. A total of 34 compounds were identified, including 12 terpenes and terpenoids, 10 aldehydes, 7 esters, 4 ketones and 1 alcohol.

Regarding the dehydrated products, it was possible to observe that, in terms of quantity and concentration of compounds, the samples submitted to the OD process presented better results for all identified classes with the exception of alcohols when compared to their respective drying controls.

It is also worth highlighting the similarity between the compound profiles of the C-FD (23 compounds) and OD-FD (25) samples, and the concentration of the compounds (mainly for aldehydes and terpenes), suggesting the efficiency of freeze-drying in preserving compounds even without pretreatment in the control sample. Freeze-drying is a drying process that aims to preserve heat-sensitive foods, which results in obtaining high-quality dried foods, with little loss of volatile compounds characterizing good organoleptic and bioactive properties [5]. A more pronounced difference was observed in the C-HAD samples, whose number of identified compounds was 20, and OD-HAD with 30 compounds.

Aldehydes represent one of the most important classes of compounds for the aroma of persimmon. It can be seen that the samples subjected to OD had a higher concentration of aldehydes compared to their respective controls and that the pretreatment had a greater influence on the HAD process, considering that the concentration of the OD-HAD sample was 2352.71 µg/kg and that of the C-HAD sample was 1191.81 µg/kg.

In a study of peach slices immersed in sucrose solution followed by freeze-drying, Wang et al. [24] found that the increase in the content of aldehyde compounds could be related to the compounds formed during the Maillard reactions.

Among the compounds of the terpene and terpenoid classes, β-cyclocitral, β-cyclohomocitral, citral, α-ionone and geranyl acetone showed greater sensitivity to the OD process followed by HAD drying compared to the C-HAD sample. However, the OD process gave the samples higher concentrations for the class in general compared to the controls. According to Torres et al. [17], OD can cause changes in the concentrations of the analyzed compounds depending on the process conditions. In a study with mango, the authors observed that volatile losses, in relation to fresh samples, were induced by the use of highly concentrated osmotic solutions and by the high level of osmohydration of the sample. Meanwhile, Zhao et al. [18] observed a trend towards an increase in volatile compounds (including some terpenes and esters) in osmotically pretreated mango samples, and that this behavior can be explained by changes in metabolic activity and fruit composition due to changes in respiration rate in samples subjected to osmotic treatment.

Wang et al. [50] identified phenylethyl alcohol in persimmon pulp and cited it as one of the aroma compounds with an impact on the fruit. In the present study, phenylethyl alcohol was identified only in control samples. As for esters, these were mainly observed in samples subjected to OD. Talens et al. [19] evaluated the effect of OD on the volatile composition of kiwi and observed that, depending on the time and sucrose concentration conditions of the pretreatment, the formation of esters and reduction in aldehydes and alcohols may occur. In addition, the authors noted that the increase in esters suggests that osmotic stress may imply an acceleration of the maturation process at the cellular level in line with enhanced enzymatic action.

Many volatile constituents of fruit come from lipid degradation. Lipolytic enzymes are released from cell compartments due to the structural collapse resulting from the dehydration process and can act on fatty acids, resulting in the release of aromatic compounds [15]. During the OD process, some compounds migrate to the osmotic solution, which may contribute to the reduction in volatile compounds [19]. Furthermore, changes in the profile and concentration of compounds may occur due to degradation-formation reactions that occur in the tissue associated with cellular stress induced by the treatment conditions [17,19].

## 4. Conclusions

The inclusion of an osmotic pretreatment to obtain oven-dried or freeze-dried persimmons proved to be an attractive process to protect the physicochemical and chemical characteristics of the fresh fruit. The highest sucrose concentration resulted in the lowest water and moisture activity values for samples dehydrated in hot air-drying. Samples subjected to the highest sucrose concentration resulted in the highest TPC and TF concentrations and the highest antioxidant activity by FRAP after freeze-drying. According to the experimental design and the Desirability function analysis, the ideal conditions established for the concentration of the sucrose solution and immersion time for the pretreatment were 29.5% and 60 min for subsequent drying in a hot air oven and 38% and 29 min for drying by freeze-drying, respectively. These standardized conditions resulted in dehydrated products with good retention of organic acids, especially succinic acid, and volatiles such as nonanal and 6-methyl-5-hepten-2-one, especially in the freeze-dried samples.

## Figures and Tables

**Table 1:** Level of independent variables values used for OD process of persimmon.

Independent Variables	Code	Unit	Factors Level				
			−1.41	−1	0	1	1.41
Sucrose	X1	%	10	20	25	30	40
Time	X2	min	20	30	40	50	60

**Table 2:** Experimental design and data from persimmon samples submitted to OD-HAD.

Assay	X_1_	X_2_	Y_1_	Y_2_	Y_3_	Y_4_
1	20	30	0.5629	12.71	109.93	83,712.86
2	30	30	0.5530	12.7	391.52	72,183.33
3	20	50	0.5540	11.00	367.46	63,355.54
4	30	50	0.5405	10.07	207.81	106,302.92
5	10	40	0.5430	11.52	296.92	77,105.19
6	40	40	0.5302	9.91	299.46	72,103.09
7	25	20	0.5576	12.74	345.83	60,572.22
8	25	60	0.5453	10.77	375.56	92,622.05
9	25	40	0.5471	10.84	239.55	63,073.17
10	25	40	0.5467	10.94	247.66	70,945.61
11	25	40	0.5439	10.92	248.78	76,749.57

OD-HAD: (osmotic dehydration with hot air-drying). X_1_: Sucrose (%); X_2_: Time (min); Y_1_: aw—water activity; Y_2_: Moisture (%); Y_3_: Total flavonoids (mg/100 g); Y_4_: ABTS (µmol/100 g).

**Table 3:** Experimental design and data from persimmon samples submitted to OD-FD.

Assay	X_1_	X_2_	Y_1_	Y_2_	Y_3_	Y_4_	Y_5_
1	20	30	391.27	1086.37	23.93	1171.07	12,065.89
2	30	30	377.68	1014.3	18.8	2986.58	142,921.42
3	20	50	354.85	767.59	14.45	2661.55	132,622.82
4	30	50	388.52	1045.84	14.98	2159.42	130,455.06
5	10	40	308.15	525.76	16.66	2430.94	148,051.56
6	40	40	1579.52	1339.91	7.61	4547.50	148,717.62
7	25	20	428.77	1111.95	31.70	1983.04	122,405.75
8	25	60	356.95	1055.75	19.81	2986.55	107,918.20
9	25	40	401.16	1122.85	16.35	1428.17	157,388.51
10	25	40	439.91	1134.07	17.95	1293.82	161,170.22
11	25	40	478.21	1126.55	19.06	1675.17	157,427.12

OD-FD: osmotic dehydration with freeze-drying. X_1_: Sucrose (%); X_2_: Time (min); Y_1_: Total phenolic compounds (mg GAE/100 g); Y_2_: Total flavonoids (mg QE/100 g); Y_3_: Total carotenoids (µg/g); Y_4_: FRAP (µmol Fe^2+^/100 g); Y_5_: ABTS (µmol TE/100 g).

**Table 4:** Predictive models and respective statistics associated with the effects of sucrose solution concentration and osmotic pretreatment immersion time in persimmons.

Dependent Variables	Predicted Values	Experimental Values	Coefficient of Variation (%)
Hot air drying			
Aw	0.5428	0.5493	0.84
Moisture (%)	11.32	10.45	5.65
Total flavonoids (mg QE/100 g)	250.72	238.18	3.63
ABTS (µmol TE/100 g)	83,000	100,000	13.14
Freeze drying			
Total phenolics (mg GA/100 g)	943.84	921.47	1.70
Total flavonoid (mg QE/100 g)	932.83	917.35	1.18
Carotenoids (µg/g)	19.66	17.93	6.51
FRAP (µmol Fe^2+^/100 g)	2859.30	2571.09	7.51
ABTS (µmol TE/100 g)	134,500	110,862	13.62

X_1_: Sucrose (%); X_2_: Time (min).

**Table 5:** Results of the organic acids contents in persimmon samples by HPLC.

Compounds	Dehydrated Persimmons—Concentrations (mg/100 g)			
	C-HAD	OD-HAD	C-FD	OD-FD
Oxalic acid	14.42 ^c^ ± 0.18	15.06 ^b^ ± 0.03	14.21 ^c^ ± 0.01	16.73 ^a^ ± 0.05
Quinic acid	15.62 ^c^ ± 0.03	17.46 ^b^ ± 0.28	15.79 ^c^ ± 0.01	18.57 ^a^ ± 0.08
L-ascorbic acid	nd	nd	0.15 ^b^ ± 0.00	2.27 ^a^ ± 0.21
Acetic acid	1.80 ^b^ ± 0.06	1.56 ^c^ ± 0.00	2.09 ^a^ ± 0.01	2.13 ^a^ ± 0.01
Citric acid	1.49 ^a^ ± 0.09	nd	1.27 ^b^ ± 0.00	1.50 ^a^ ± 0.02
Succinic acid	2.13 ^c^ ± 0.18	8.38 ^a^ ± 0.02	1.26 ^d^ ± 0.02	5.96 ^b^ ± 0.02
Fumaric acid	0.24 ^b^ ± 0.00	0.25 ^a^ ± 0.00	nd	nd

C-HAD: Control persimmon (without osmotic dehydration) and hot air-drying; OD-HAD: persimmon with osmotic dehydration (best condition of the experimental design) and hot air-drying; C-FD: Control persimmon (without osmotic dehydration) and freeze-drying, and OD-FD: persimmon with osmotic dehydration (best condition of the experimental design) and freeze-drying. nd: Concentration below detection limit; Results are expressed as mean ± standard deviation (n = 3). Means followed by the same letter in the rows do not differ by Tukey’s test at the 5% significance level (*p* ≤ 0.05).

**Table 6:** Volatile compounds of dehydrated persimmons.

N°.	Compounds	CAS	LRI_E_	LRI_L_	Dehydrated Persimmons—Concentrations (µg/kg) *				Odor
					C-HAD	OD-HAD	C-FD	OD-FD	
	Aldehydes								
1	(*E*)-2-Hexenal	6728-26-3	859	856	nd	nd	141.33 ± 2.29	187.82 ± 6.52	Green
2	Heptanal	111-71-7	901	903	nd	143.91 ± 19.67	nd	nd	Green, fresh
3	Benzaldehyde	100-52-7	956	960	226.30 ± 22.30	148.31 ± 10.03	199.23 ± 6.52	212.68 ± 4.85	Sweet, cherry
4	Octanal	124-13-0	1002	1004	nd	238.73 ± 6.78	230.51 ± 4.18	260.15 ± 11.56	Green, citrus
5	(*E*)-2-Octenal	2548-87-0	1056	1056	nd	278.74 ± 4.18	239.25 ± 4.82	301.56 ± 0.40	Green, citrus
6	Nonanal	124-19-6	1103	1104	569.70 ± 13.56	707.30 ± 25.91	578.22 ± 2.40	759.12 ± 13.15	Rose, fresh
7	(*E*)-2-Nonenal	18829-56-6	1158	1158	nd	145.31 ± 1.74	71.36 ± 2.11	142.49 ± 4.72	Green, cucumber
8	Benzaldehyde, 2.5-dimethyl-	5779-94-2	1169	1164	116.14 ± 5.98	103.62 ± 7.33	97.71 ± 3.80	107.40 ± 3.32	
9	Decanal	112-31-2	1199	1200	239.03 ± 4.17	494.57 ± 24.03	503.82 ± 14.10	543.00 ± 7.09	Green, cucumber
10	Dodecanal	112-54-9	1407	1407	40.64 ± 0.73	92.23 ± 6.89	111.07 ± 0.51	149.31 ± 4.46	Citrus, floral
	Subtotal				1191.81	2352.71	2172.51	2663.5	
	Alcohols								
11	Phenylethyl Alcohol	60-12-8	1112	1112	191.21 ± 7.52	nd	260.12 ± 39.53	nd	Floral, sweet, rose
	Subtotal				191.21	0.00	260.12	0.00	
	Ketones								
12	6-Methyl-5-hepten-2-one	110-93-0	984	982	603.88 ± 22.00	770.91 ± 34.70	535.40 ± 21.54	1065.92 ± 270.67	Citrus, fruity
13	2,6,6-Trimethylcyclohexanone	2408-37-9	1030	1034	365.83 ± 4.51	467.18 ± 7.75	nd	nd	Pungent
14	3,5-Octadien-2-one	38284-27-4	1090	1074	nd	221.80 ± 0.08	160.58 ± 5.15	197.03 ± 3.15	Fruity
15	2-Cyclopentylcyclopentanone	4884-24-6	1276	-	72.76 ± 0.13	120.70 ± 5.97	105.41 ± 3.31	138.69 ± 3.40	Fruity, mint
	Subtotal				1042.48	1580.59	801.39	1401.63	
	Terpenes and terpenoids								
16	D-Limonene	5989-27-5	1024	-	nd	162.63 ± 4.61	nd	nd	Citrus, orange, sweet
17	Citronellal	106-23-0	1151	1149	nd	286.80 ± 5.68	nd	nd	Rose, green
18	Safranal	116-26-7	1193	1195	180.82 ± 9.04	239.73 ± 8.47	345.02 ± 9.79	387.43 ± 4.66	Aldehydic, sweet
19	Verbenone	80-57-9	1208	1206	nd	nd	147.04 ± 8.24	151.18 ± 2.03	Menthol
20	β-Cyclocitral	432-25-7	1214	1208	257.08 ± 13.73	172.40 ± 36.79	224.75 ± 10.46	163.00 ± 17.07	Herbal. saffron
21	β-Citral	106-26-3	1231	1240	24.44 ± 1.02	38.65 ± 0.73	nd	44.01 ± 1.92	Citrus, sweet
22	β-Cyclohomocitral	472-66-2	1246	1251	216.33 ± 8.00	150.20 ± 8.74	120.37 ± 3.70	223.25 ± 4.01	Citrus, woody
23	Citral	5392-40-5	1263	1239	60.65 ± 4.45	54.24 ± 1.94	nd	nd	Citrus, sweet
24	α-Ionone	127-41-3	1414	1412	122.24 ± 0.11	89.08 ± 8.17	134.20 ± 4.88	220.54 ± 1.67	Floral, violet
25	Geranyl acetone	3796-70-1	1442	1447	348.42 ± 17.92	248.23 ± 14.76	315.57 ± 13.31	353.25 ± 5.53	Floral, fruity
26	β-Ionone	14901-07-6	1468	1480	531.16 ± 33.48	638.59 ± 16.17	533.29 ± 27.48	748.56 ± 46.78	Floral, fruity
27	β-Ionone epoxide	23267-57-4	1471	1455	65.80 ± 3.19	nd	247.53 ± 6.81	138.32 ± 3.66	Fruity, sweet
	Subtotal				1806.94	2080.56	2067.77	2429.53	
	Esters								
28	Methyl octanoate	111-11-5	1123	1123	nd	166.40 ± 6.08	nd	nd	Green, fruity
29	Ethyl octanoate	106-32-1	1196	1196	51.53 ± 4.43	262.72 ± 7.68	129.14 ± 1.89	312.93 ± 5.35	Sweet, wine
30	Ethyl nonanoate	123-29-5	1295	1294	nd	32.01 ± 0.02	nd	nd	Fruity, wine
31	Ethyl decanoate	110-38-3	1394	1394	nd	116.98 ± 6.73	nd	164.11 ± 6.70	Fruity, sweet
32	Methyl hexadecanoate	112-39-0	1924	1926	nd	63.59 ± 6.29	nd	nd	Oily, greasy
33	Ethyl 9-hexadecenoate	54546-22-4	1970	1975	nd	92.32 ± 2.73	nd	92.46 ± 3.83	
34	Ethyl hexadecanoate	628-97-7	1992	1994	18.26 ± 1.12	67.95 ± 9.98	52.21 ± 1.80	85.93 ± 1.35	Fermented, fruity
	Subtotal				69.79	801.97	181.35	655.42	

* Values expressed as mean ± standard deviation. LRIE: Linear retention index on HP-5MS obtained experimentally; LRIL: Linear retention index from literature (https://webbook.nist.gov/; accessed on 26 April 2024). C-HAD: Control persimmon (without osmotic dehydration) and hot air-drying; OD-HAD: persimmon with osmotic dehydration (best condition of the experimental design) and hot air-drying; C-FD: Control persimmon (without osmotic dehydration) and freeze-drying; and OD-FD: persimmon with osmotic dehydration (best condition of the experimental design) and freeze-drying. nd: Concentration below detection limit.

## Data Availability

The original contributions presented in the study are included in the article, further inquiries can be directed to the corresponding author.
